# The Effect of Acute Hyperglycaemia Induced by Oral Glucose Load on Heart Rate Variability and Skin Microvascular Reactivity in Young Adults

**DOI:** 10.3390/life14010056

**Published:** 2023-12-28

**Authors:** Jernej Šorli, Helena Lenasi

**Affiliations:** 1 General Hospital Dr. Franc Derganc, 5290 Šempeter pri Gorici, Slovenia; 2 Institute of Physiology, Faculty of Medicine, University of Ljubljana, Zaloška 4, 1000 Ljubljana, Slovenia

**Keywords:** hyperglycaemia, oral glucose tolerance test, heart rate variability, sympathetic nervous system, insulin, laser Doppler fluxmetry, microcirculation, endothelium, post-occlusive reactive hyperaemia, glucose tolerance

## Abstract

We aimed to elucidate the effects of acute hyperglycaemia, induced by an oral glucose tolerance test (OGTT), on the autonomic nervous system (ANS) and skin microvascular reactivity at the time point of peak plasma glucose concentration (c_glc_) in 20 young, healthy participants. We assessed their heart rate variability (HRV) as a measure of the ANS activity and the parameters of post-occlusive reactive hyperaemia (PORH) to estimate skin microvascular reactivity as measured by laser Doppler (LD) fluxmetry. The tests were repeated 30 min after a standard OGTT (75 g glucose dissolved in 250 mL water) and, in a separate control experiment, after drinking the same amount of water. Participants had their c_glc_ and serum insulin measured at three consecutive time-points according to the testing protocol. The low-frequency (LF) spectral power, the LF to high-frequency (LF/HF) ratio, and the diastolic blood pressure increased significantly more after water than after OGTT, and there was a trend of the peak LD flux of PORH decreasing more after OGTT than after water. Significant correlations between some PORH and all the HRV parameters and c_glc_ increase after OGTT were found, implying diminished vascular reactivity evoked by hyperglycaemia in healthy subjects with lower glucose tolerance.

## 1. Introduction

The long-term effects of chronic hyperglycaemia on vascular integrity are well known, with the principal pathogenic mechanism being (micro)vascular endothelial dysfunction [1,2]. In endothelial cells, hyperglycaemia has been shown to cause mitochondrial hyperpolarization and endothelial nitric oxide synthase (eNOS) uncoupling, thereby increasing reactive oxygen species (ROS) and decreasing endothelial vasodilators production as well as activating inflammatory pathways, which impairs endothelial vasodilatory capacity [1,2,3]. According to in vitro studies, it usually takes hours to days for ROS production to increase, with evidence suggesting alterations in RNA expression [3,4,5]. On the other hand, decreased vasodilator production was observed after only 20 min incubation in high-glucose solutions in some experimental settings; however, the effect was significant at unphysiologically high glucose concentrations (higher than 25 mM) and was absent at lower concentrations [5,6].

Interestingly, acute hyperglycaemia has been shown to impair microvascular dilation in humans in vivo on different timescales, ranging from 20 min to three hours, depending on experimental settings [7,8,9,10,11,12,13,14], although with several opposing findings [15,16,17,18,19,20,21]. In some studies, even an improvement of vascular function during exposure to acute hyperglycaemia was observed [18,20]. Several authors suggested that the endothelium-dependent vasodilation is impaired only in states of deranged insulin sensitivity and not in healthy subjects [14,16,19,22,23]. The discrepant findings imply that in vivo, the mechanisms are far more complex, and there might be other endothelium-dependent and -independent mechanisms altering the vasodilatory capacity during acute hyperglycaemia.

One possible mechanism affecting the vascular vasodilatory potential might be mediated by insulin, which was shown to increase endothelial vasodilator production in various settings in vitro [24,25,26,27]. The action of insulin on endothelial cells was observed in seconds following insulin administration, rendering the glucose- and insulin-counteracting hypothesis viable. Moreover, there is evidence for the in vivo effects of insulin on microcirculation: Iredahl et al. showed that transdermal application of insulin induced endothelium-dependent vasodilation in skin microcirculation in healthy subjects, presumably mediated by nitric oxide (NO) [28], while Rossi et al. implicated its effect on the vascular smooth muscle [29]. In addition, insulin was implicated in the increase in functional capillary density, which has been shown to directly correlate with insulin sensitivity in human skin microcirculation, reinforcing the idea that capillary recruitment plays an important role in enhancing insulin-mediated glucose uptake [30].

Another mechanism by which acute hyperglycaemia affects vascular reactivity might be mediated by the autonomic nervous system (ANS). Both glucose and insulin have been shown to alter the ANS activity in humans [18,31,32,33,34,35,36,37,38,39], although with some opposing findings [20,40,41,42].

It has not been thoroughly evaluated how these to some extent contradictory mechanisms integrate at the level of skin microcirculation, which has a complex anatomical and functional organization [43,44,45]. While microvasculature in all skin areas is innervated by the vasoconstrictor fibres of the sympathetic nervous system (SNS), the non-glabrous, non-acral skin areas also receive the sympathetic vasodilatory fibres. However, the noradrenergic vasoconstrictor response is the most powerful in glabrous acral parts with abundant arteriovenous anastomoses (AVAs), i.e., special anatomical structures that are not present in other skin sites [43,44,45]. Other control mechanisms include hypoxic metabolites, intrinsic myogenic activity, axon reflex mediated, and, most importantly, endothelium-dependent mechanisms, with different contributions thereof to the regulation of vascular tone in various sites [43,44]. The abundance of different mechanisms involved in the regulation of microvascular blood flow makes an evaluation of a particular mechanism separately from the others difficult. Moreover, due to large spatial and temporal variability, rather than measuring resting blood flow, vascular reactivity should usually be assessed [44,46].

In light of that mentioned above, our aim was to investigate the impact of acute hyperglycaemia induced by an OGTT on microvascular function in young, healthy volunteers at the time point of peak plasma glucose concentration (c_glc_). To this end, we assessed skin microvascular reactivity by inducing post-occlusive reactive hyperaemia (PORH), a potential measure of endothelial function, and performed heart rate variability (HRV) analysis to assess ANS. Additionally, we measured plasma glucose and insulin concentration at particulate time points before and after OGTT. Based on animal model studies and some previous reports, we hypothesised that vascular reactivity would be diminished after OGTT, potentially reflecting impaired endothelium-dependent vasodilation and/or alterations in the activity of the ANS.

## 2. Materials and Methods

### 2.1. Participants

Twenty healthy volunteers (15 males, 5 females) aged 21.6 years ± 1.6 years, non-smokers, and all with a normal BMI were invited to undergo the study protocol (ingestion of high-glucose solution dissolved in water) and the control protocol (ingestion of water) on two separate occasions. Female participants were confirmed to have been in the follicular phase of the menstrual cycle. The participants were asked to attend the trial in a fasting state (after having consumed “normal” diet in the last week before the measurements), to ingest nothing but water in small amounts 12 h prior to arrival to the laboratory, and to avoid strenuous exercise 24 h before the trial; none of them had been taking any medications.

The study was approved by the National Medical Ethics Committee (No. 0120-175/2017/6) and conducted in accordance with the principles of the Declaration of Helsinki and the European Convention on Human Rights and Biomedicine. Written informed consent was obtained from the participants before starting the experiment.

### 2.2. Skin Perfusion and Reactive Hyperaemia Assessment

Skin perfusion was assessed by means of laser Doppler (LD) fluximetry, using Periflux 4001 Master/4002 Satellite system (Perimed, Järfälla, Sweden). In short, the method is based on the Doppler shift of the laser light penetrating approximately 1 mm in depth and reflected from moving erythrocytes and measured by a probe attached to the skin [43]. The depth corresponds to the deep subpapillary microvascular layer consisting of arterioles, capillaries, venules, and arteriovenous anastomoses (AVAs); LD flux is linearly related to blood flow in skin microcirculation and expressed in arbitrary perfusion units (PU) [43]. After zero-calibration, LDF was measured in baseline, resting conditions; afterwards, a 3 min occlusion of the brachial artery was performed and the parameters of PORH assessed to test vascular reactivity. Although many mechanisms contribute to the phenomenon of PORH [43,47], PORH has been used in clinical practice to assess the endothelial function. As skin microvascular flow is controlled by different mechanisms partly depending on the measuring site, we measured skin blood flow in two corresponding sites: on the volar forearm (non-glabrous, non-acral site) and on the finger pulp, which is a glabrous acral site with abundant AVAs.

### 2.3. Heart Rate Variability Analysis

The contribution of the sympathetic and the parasympathetic component of the ANS to the regulation of the heart rate (HR) can be evaluated by heart rate variability (HRV) analysis [48,49,50,51]. We performed frequency spectral analysis of the R-R interval in 10 min ECG recordings in resting conditions using NeuroKit software package [52]. Low-frequency (LF) spectral power and high-frequency (HF) spectral power were determined from frequencies between 0.04 Hz–0.15 Hz and 0.15 Hz–0.40 Hz, respectively, which are believed to reflect baroreceptor-reflex-dependent (LF) and respiration-dependent (HF) cardiovagal and SNS-mediated HRV [48,53].

### 2.4. Protocol

All measurements were performed in the morning in a temperature-controlled room (23 °C). After arrival at the laboratory, participants underwent a 30 min acclimatization period in a supine position.

#### 2.4.1. Plasma Glucose Measurement

The participants had their capillary blood samples taken from the right index finger to assess their c_glc_ spectrophotometrically using HemoCue^®^ Glucose 201+ (HemoCue AB, Ängelholm, Sweden). Blood samples were obtained three times: upon arrival prior to any measurements, 30 min after OGTT/control trial (before the start of the haemodynamic measurements), and at the end of haemodynamic measurements. The time was chosen based on a previous pilot experiment designed to define the time when c_glc_ reached its peak value.

#### 2.4.2. Measurement of the Haemodynamic Parameters

Throughout the protocol, a three-channel ECG was recorded for continuous HR measurement. A digital inflatable cuff was placed on the proximal phalanx of the right middle finger for continuous digital artery blood pressure (BP) measurement (Finapress^TM^ BP monitor, Ohmeda 2300, Englewood, CO, USA). Double adhesive LD probes were placed on the volar site of the left forearm and on the left middle finger pulp. The participants were asked to remain in the same comfortable position throughout the experiment and avoid any movements. All haemodynamic variables were measured in the following manner:

- 10 min baseline period;
- 3 min supra-systolic occlusion of the brachial artery;
- 10 min after the release of the occlusion (normalisation period).

Following the above procedure, the participants underwent a standard OGTT: They were asked to drink 75 g glucose dissolved in 250 mL water in a five-minute period. Thirty minutes after glucose ingestion, c_glc_ was determined and the haemodynamic measurements performed as described.

The same protocol was repeated on a separate occasion under the same experimental conditions as a control trial, when participants ingested 250 mL water instead of glucose solution.

### 2.5. Glucose and Insulin Kinetics

A separate trial was performed to assess plasma glucose and insulin kinetics before (fasting state, time 0) and 30, 50 (corresponding to the start and the end of the haemodynamic measurements, respectively), and 90 min after OGTT. Each participant had an intravenous cannula inserted in a forearm peripheral vein and venous blood (VB) samples collected in pairs: the insulin sample (test tube for serum preparation) immediately after the glucose sample (anticoagulant-coated tube). All test tubes were centrifuged and placed on ice immediately after collection. Serum insulin concentration (c_ins-VB_) was determined by the commercially available solid-phase, enzyme-labelled chemiluminescent immunometric assay, using the “Immulite2000 Insulin” kit and Immulite2000-XPI analyser (both Siemens Healthcare Diagnostics Products Ltd., Camberley, UK). For quantitative determination of plasma glucose (c_glc-VB_), the enzymatic UV test (hexokinase method) was used (Olympus AU400 system; Mishima Olympus CO., Ltd., Tokyo, Japan; reagent: Beckman Coulter, Inc., Kildare, Ireland).

### 2.6. Data Acquisition and Statistical Analysis

Haemodynamic data (with a sampling rate of 500 Hz) were acquired and processed by Nevrokard software package (https://www.nevrokard.eu/contact.htm, accessed on 22 December 2023) (Nevrokard Kiauta, d.o.o., Izola, Slovenia).

Time-averaged systolic (SP), mean (MP), diastolic (DP) and pulse (PP) pressure, HR (expressed reciprocally as R-R interval—t_RR_), resting LDF (LD_rest_), LF, HF, and LF/HF ratio were determined from the initial 10 min resting interval preceding each trial. As for the PORH assessment, the peak post-occlusion LD—LD_peak_, the time interval from the occlusion release to LD_peak_—t_peak_, the area under the curve of PORH—AUC, and the time-averaged LD flux of the normalization period—LD_base_ were determined from PORH curves for both skin sites.

For the systemic error control, all the initial values (pre-OGTT and pre-water) were compared by Mann–Whitney test. Variables were then compared between OGTT and water (control) as pre-post treatment changes (Δvariable = post-treatment − pre-treatment) using Wilcoxon test. PORH and HRV parameters changes following OGTT were divided into two groups (of comparable sample size) depending on Δc_glc_ 30 min after OGTT—“low glucose” (LG) group (LG, Δc_glc_ < 2.3 mM) and “high glucose” (HG) group (HG, Δc_glc_ ≥ 2.3 mM). LG, HG, and post-water changes were compared to each other using ANOVA (post hoc Tukey). The haemodynamic variables (LDF, PORH, and HRV) were correlated with Δc_glc_ using univariate linear regression.

Consecutive c_glc_ and c_ins_ obtained in the second glucose and insulin kinetics trial were analysed in the corresponding time points after OGTT using repeated-measures ANOVA (post hoc Tukey). Maximal c_glc-VB_ and c_ins-VB_ were compared using linear regression.

Variables are presented as median and interquartile range (Q_1_–Q_3_) or mean ± SD where appropriate. Normality of distribution was checked using Shapiro–Wilk test. For all statistical tests, the significance level of 0.05 applies, and 95% confidence interval (CI) is given. Statistical analysis and graphical representation of data were carried out using Jamovi statistical package [54].

## 3. Results

**Figure 1:**
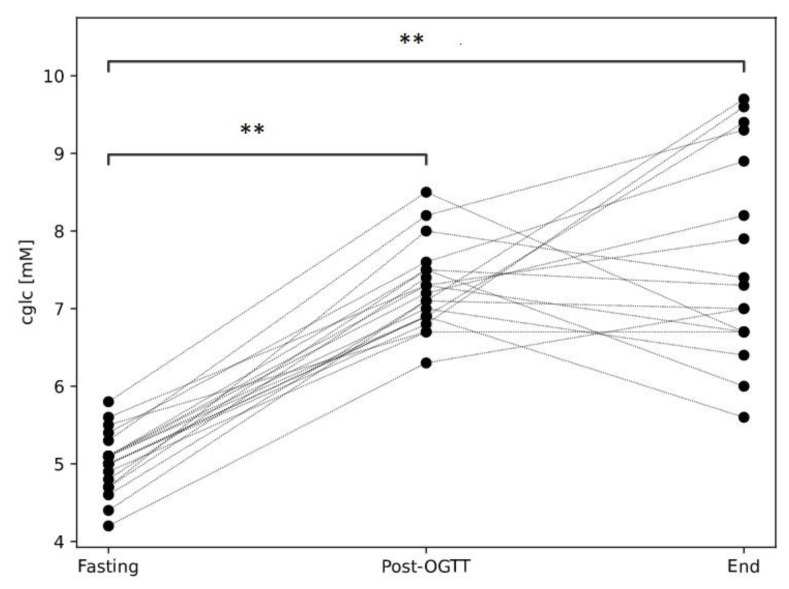
Plasma glucose concentration (c_glc_) determined in capillary blood sample before and after OGTT. Fasting, before OGTT; Post-OGTT, 30 min after OGTT; End, at the end of the protocol (i.e., 50 min after OGTT). ** *p* ˂ 0.001, paired *t*-test, with respect to fasting.

Fasting c_glc_ (before any provocation) obtained from the capillary blood sample was 5.0 mM ± 0.4 mM pre-OGTT and 5.2 mM ± 0.4 mM pre-water; no significant difference was found between pre-OGTT and pre-water (CI: −0.4 mM–0.1 mM, *p* = 0.123, independent sample *t*-test). Further, 30 min after the provocation (OGTT and water, respectively), c_glc_ increased for 2.2 mM ± 0.5 mM after OGTT and 0.0 mM ± 0.5 mM after water (CI: 1.8 mM–2.58 mM, *p* = 0.000, paired sample *t*-test). The increase in c_glc_ at three consecutive time points throughout the OGTT protocol is presented in Figure 1. The data of c_glc_ after the control trial with water are not shown, as c_glc_ did not exhibit any significance with respect to the fasting value.

Baseline values of the haemodynamic variables before any provocation (either OGTT or water) are summarised in Table 1. It is worth mentioning that the haemodynamic variables did not differ pre-OGTT compared to pre-water justifying subsequent comparison of the changes evoked by treatment. Moreover, PORH had no effect on BP and HR.

### 3.1. Haemodynamic Changes after OGTT and Water

The changes of some haemodynamic variables were significantly different following OGTT compared to water and are presented in Table 2. Glucose and water induced an increase in systolic and diastolic BP and had no effect on the HR, yet only the diastolic BP increment was significantly larger after water compared to glucose. Also, LF and LF/HF increased significantly more after water than after OGTT, and there was a trend of LD_peak_ decreasing more after OGTT than after water (Table 2) in the finger pulp.

### 3.2. Dependence of Haemodynamic Changes on Plasma Glucose Concentration Increase after OGTT

**Figure 2:**
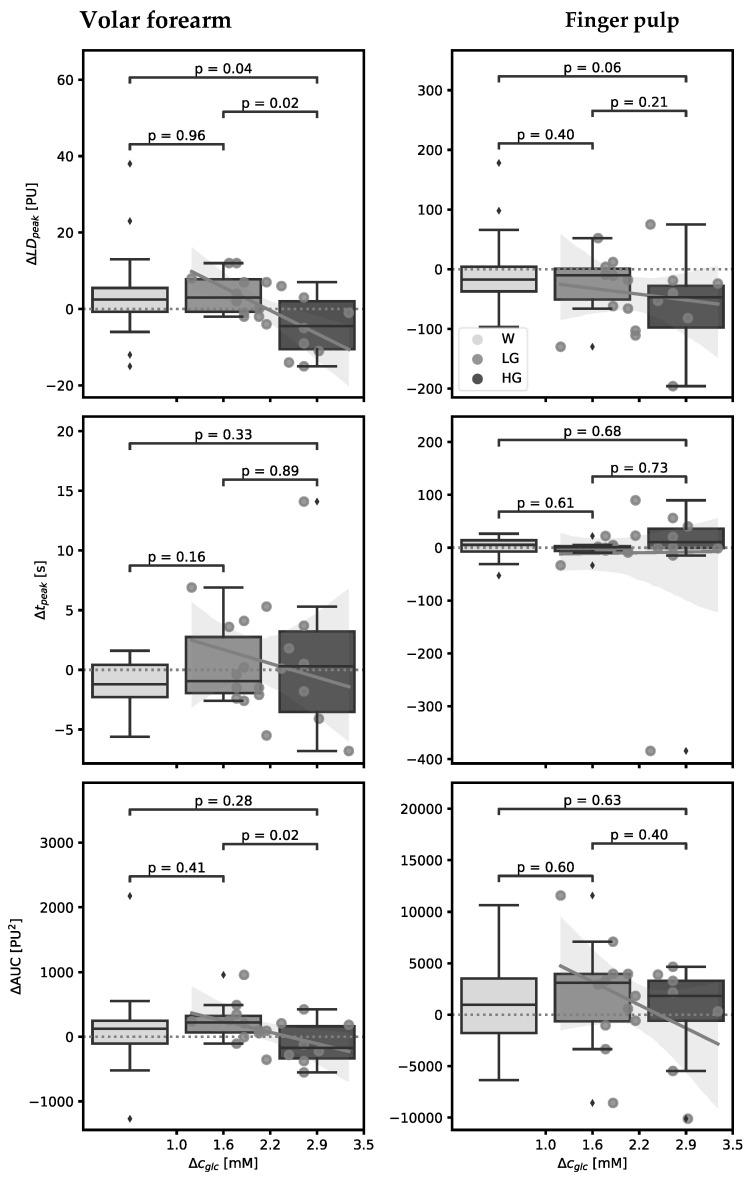
The post-occlusion reactive hyperaemia (PORH) parameters change (Δ) correlated to increase of glucose concentration assessed from capillary blood (Δc_glc_) after oral glucose tolerance test (OGTT) (dark bars) and water load (W, control, the first—bright bar) on the forearm (**left** plots) and on the finger pulp (**right** plots), with the regression line. Middle bar represents the low-glucose (LG) group (Δc_glc_ < 2.3 mM); the bar on the right (the darkest colour) represents the high-glucose (HG) group (Δc_glc_ ≥ 2.3 mM). ΔLD_peak_, pre-post glucose/water load change in peak laser Doppler flux; Δt_peak_, pre-post post glucose/water load change in time to LD_peak_; ΔAUC, pre-post glucose/water load change in the area under the hyperaemic response curve; PU, perfusion unit; *p*, *p*-values of ANOVA, post hoc Tukey.

**Figure 3:**
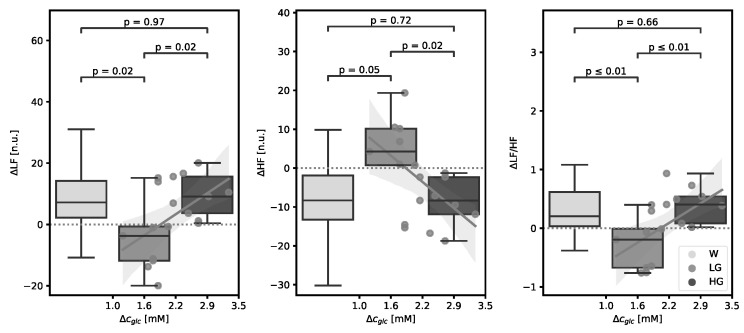
The heart rate variability (HRV) parameters change (Δ) relative to glucose concentration increase (Δc_glc_) after oral glucose tolerance test (OGTT) and water load (W, control, left bar) with the regression line. Middle bar represents the low-glucose (LG) group (Δc_glc_ < 2.3 mM); the bar on the right (the darkest colour) represents the high-glucose (HG) group (Δc_glc_ ≥ 2.3 mM). ΔLF, pre-post glucose/water load change in low-frequency band of HRV; ΔHF, pre-post glucose/water change in high-frequency band of HRV; ΔLF/HF, pre-post glucose/water load change in low- to high-frequency band ratio of HRV; n.u., normalised unit; *p*, *p*-value of ANOVA, post hoc Tukey.

We found significant correlations between some PORH and all the HRV parameters and Δc_glc_ (Figure 2 and Figure 3), with the standardised regression coefficients and the corresponding *p*-values of Pearson’s test presented in Table 3.

According to post-OGTT Δc_glc_, we divided subjects into “low glucose” (LG) and “high glucose” (HG) groups, respectively (Δc_glc_ < 2.3 mM for LG and ≥2.3 for HG), and those changes were compared between both groups and to the changes following water load (Figure 2 and Figure 3). Changes in PORH parameters on the volar forearm were greater in the HG group and were of opposite direction compared to the ones in the LG group and the ones after water load, whereas changes on the finger pulp exhibited the same directions in the HG and LG groups, respectively, and showed some dose dependence (Figure 2). Summarizing, on the volar forearm, ΔLDF_peak_ significantly negatively correlated with Δc_glc_, whereas in the pulp, negative correlations between ΔLDF_peak_ and ΔAUC and Δc_glc_ did not reach statistical significance. HRV parameters showed consistent dynamics of changes between groups, with the HG group exhibiting similar changes to the ones after water load and opposite changes to the LG group (Figure 3). LF (similar as LF/HF ratio) was positively, and HF negatively correlated to Δc_glc_. The division of the results into two groups regarding post-OGTT Δc_glc_ seems reasonable, as the last Δc_glc_ obtained after the end of haemodynamic measurements exhibited significant scattering, compromising a uniform interpretation of the data.

### 3.3. Glucose and Insulin Kinetics

Fasting c_glc-VB_ and c_ins-VB_ were 4.3 mM ± 0.3 mM and 2.6 µUml^−1^ ± 1.9 µUml^−1^, respectively. Both c_glc-VB_ and c_ins-VB_ increased after OGTT, as shown in Table 4. Maximal c_glc-VB_ and c_ins-VB_ were positively correlated (Pearson regression coefficient r = 0.59; *p* ≤ 0.05). Worth noting is that glucose concentration of the venous blood samples exhibited similar values to the ones obtained from the capillary blood.

## 4. Discussion

To the best of our knowledge, this is the first study simultaneously assessing vascular reactivity and SNS activity noninvasively while providing evidence that the measured changes occurred during peak c_glc_, i.e., 30 min after an oral glucose load. Our results imply that OGTT affects SNS activity, as assessed by HRV analysis in healthy young humans and modestly impairs vascular reactivity assessed by inducing PORH in skin microcirculation, albeit less conclusive. Both SNS activity and vascular reactivity changes were found to significantly depend on the magnitude of Δc_glc_ after OGTT, a possible measure of glucose tolerance. The findings might help elucidate the physiological changes following orally administered glucose load on one hand and address the discrepancies between existing publications in the field on the other.

As for the effect of glucose and insulin on the activity of the SNS, several authors observed that both high c_glc_ or c_ins_ administered intravenously increased SNS activity in healthy humans [18,31,32,36,37,38,40] and altered the baroreflex sensitivity [41,55]. Based on our findings, we believe that after an oral administration of glucose, such as in OGTT, this effect might be counterbalanced by vagal reflexes due to carbohydrate content in the gastrointestinal tract and, possibly, anticipation [23,56]. However, some studies performed in healthy participants where glucose was administered orally also observed increased SNS activity [34,39,57] and partly attributed it to the effect of insulin, which contrasts with our study. Firstly, a possible explanation for the discrepancy might be that the participants in those studies were 10–80 years older than our participants, which could imply different glucose tolerance and/or different ANS response. The relationship between age and glucose tolerance has been well established as well as the relationship between glucose tolerance and ANS response to hyperglycaemia and hyperinsulinemia [40,41]. In the studies mentioned above, where glucose was administered orally, the c_glc_ after the load was significantly higher compared to our study, implying a higher glucose tolerance among our participants who were indeed much younger and very homogenous in age, which was not the case in many available studies. Positive correlations between LH (and LF/HF) and Δc_glc_ after OGTT in our study imply glucose’s effect on the SNS. Additionally, we observed a decrease in SNS activity in the LG group, while there was no change in the HG group, supporting the glucose tolerance conjecture. If glucose tolerance was indeed higher in the LG group, as supported by the results correlating c_glc-VB_ or c_ins-VB_ in our study, it is possible that other studies failed to observe potential SNS attenuation because of lower glucose tolerance in older participants [34,39,57]. Moreover, SNS activity has been correlated to insulin resistance in many independent studies [27,38,39].

Secondly, our study is unique in terms of the time after OGTT when we assessed the parameters of HRV. According to glucose kinetics studies, plasma glucose and intercellular glucose concentration in skeletal muscles and subcutaneous tissue are balanced rapidly, with a maximum delay of 8 min [58,59]. Moreover, insulin-induced capillary recruitment increases glucose uptake by the tissues [30,60]. Based on our pilot experiment, we were able to determine the time interval of the peak c_glc_ and performed the subsequent haemodynamic measurements in this time frame (30 min after OGTT). In the above studies [34,39,57], the SNS activity was assessed at least one hour after OGTT. It is possible that at that time, the aforementioned vagal response diminishes, and the sympathetic dominance prevails. In one study performing OGTT and another using intravenous infusion of insulin to obtain physiological c_ins_, they repeated the SNS activity measurement immediately after c_glc_ normalisation and showed that the SNS activity returned to normal by then [31,39]. On the other hand, using unphysiologically high c_ins_ (70–150 µU/mL) or keeping c_ins_ elevated for a longer period (2 h) induced a prolonged increase in SNS activity even after normalization of c_glc_ [31,32]. Therefore, physiological elevation of c_glc_ and c_ins_ in healthy humans, at least the ones with high glucose tolerance, should be expected to coincide with SNS activity changes. Moreover, when experimentally inducing hyperinsulinemia by a continuous infusion of insulin (with and without glucose clamping), Berkelaar et al. [40] as well as Schroeder et al. [61] were not able to unequivocally confirm an impact on the vagal tone, yet the mode of inducing hyperinsulinemia in their studies was quite different and indeed importantly differed from the physiological conditions simulated in our study. On the other hand, Horton et al. showed that potential detrimental effects of high glucose were blunted by insulin’s vasoactive actions despite insulin effects on the SNS [62], yet their results could not be directly compared to our study, as they assessed microvascular response in the skeletal muscle microvasculature, which might behave differently from cutaneous microcirculation. Anderson et al. also showed that hyperinsulinemia induced by insulin infusion induced SNS activation and a concomitant increase in forearm blood flow as assessed by venous occlusion plethysmography [32]. Nevertheless, the effects of insulin on the ANS should be differently interpreted when considering the effects on macro- and microvasculature and the heart or on the central nervous system [37,55].

Moreover, different responses in various studies might be explained by the time lapse between the peak concentration of c_glc_ and c_ins_, respectively. In this time frame, the contribution of glucose and insulin to vascular reactivity and the activity of the ANS might vary.

Aside from potential effects on the SNS affecting microcirculation, combined effects of acute hyperglycaemia and hyperinsulinemia on other, locally mediated mechanisms and the endothelial component regulating skin microcirculation should be considered when referring to microvascular reactivity, at least in the time frame in which we performed measurements, keeping in line with the known effects of glucose on endothelial function [1,3,7,27]. Interestingly, our results showed inconclusive evidence regarding vascular reactivity following OGTT in general, but an evident forearm vascular reactivity decrease in the HG group. Similarly, as for the impact of OGTT on the SNS activity elucidated above, we believe that the effect of acute hyperglycaemia on skin microcirculation depends on glucose tolerance, assumingly impairing vascular reactivity in subjects exhibiting lower glucose tolerance without an effect in those with high glucose tolerance, which fits well into the assumption of optimal glucose uptake in high insulin sensitivity [30]. Indeed, a positive correlation between both the LF spectrum and the LF/HF ratio (reflecting SNS activity) and Δc_glc_ and a negative correlation between the forearm peak LDF and AUC and Δc_glc_ after OGTT in our study imply deranged cutaneous microvascular control in subjects with lower glucose tolerance. In higher glucose tolerance, the integrated effect of OGTT on SNS and local and endothelium-derived factors remains unexplained. It may be that the attenuated SNS activity or a direct vasoactive insulin action balance potentially negative effects of OGTT on local and endothelial factors. This may be the reason why in some well-controlled studies, no changes of vascular reactivity after OGTT were noticed [17,19,21]. Indeed, Natali et al. concluded that potentially negative effects of acute hyperglycaemia on endothelial function (as assessed by iontophoresis of acetylcholine) might be counterbalanced by direct vascular effects of hyperinsulinemia in healthy subjects [16]. Another possibility is that acute hyperglycaemia does not affect microvascular reactivity in subjects with higher glucose tolerance (i.e., higher insulin sensitivity).

As several studies found no vascular reactivity alterations either after OGTT or after intravascular glucose infusion of various durations [14,15,16,17,18,19,20,21,22,23], the included subjects might have had higher glucose tolerance/insulin sensitivity, which was not assessed in these studies. Nevertheless, the results of the studies are discrepant, as some did show different vascular reactivity response depending on glucose tolerance [14,16,22,23], while others even reported an impaired vascular reactivity in healthy subjects without addressing glucose tolerance [7,8,9,10,11,12,13,19]. Lower glucose tolerance in some studies could be due to included participants who were at least ten years older compared to our participants [8,9,10,35]. The only comparable study regarding the age of the included cohort as well as similar protocols to test microvascular reactivity did show an impairment of PORH but did not assess the parameters of HRV to test the ANS or correlate the glucose change to the variables assessed [7].

Moreover, many studies lacked a control experiment, which we believe is necessary, as the control trial with water in our study invoked some changes of haemodynamic and HRV parameters. In addition, the direct comparison between the studies is questionable, as the time elapsed between oral glucose load and vascular reactivity testing differed, with most studies assessing vascular reactivity one hour after glucose load or even longer. Moreover, many studies used different approaches to test vascular reactivity, which renders direct comparison questionable.

It might also be that potentially detrimental effects of glucose need a certain time frame for glucose to act on intracellular mechanisms, such as an increase in oxidative stress, increase in protein kinase C activity, and NOS uncoupling, all decreasing the endothelial vasodilator capacity [1,2,3,30] and implying a need to more systematically assess different times of high-glucose exposure on microvascular (endothelial) function.

Finally, it should be noted that aside from affecting the ANS, oral consumption of nutrients also challenges some other gastrointestinal hormones, which, besides metabolic and hormonal influences, have also been implicated in inducing indirect and potentially direct vasoactive effects [63,64]. Moreover, there are implications of different responses regarding glucose uptake and metabolic and vasodilatory effects of insulin depending on the mode of insulin action, i.e., a physiological increase after an OGTT or applied locally per micro-dialysis [60]. These observations additionally support the complex interplay of various players and hamper solid conclusions.

In the present study, we observed no differences in vascular reactivity between the LG group and the control. Therefore, we did not provide sufficient evidence for any detrimental effects on local and endothelial factors controlling skin microcirculation in subjects with higher glucose tolerance, whereas the effect in lower glucose tolerance was obvious, which exposes a need to establish surrogate markers to test glucose tolerance also in the healthy. Assessing serum insulin concentration after OGTT might be regarded as an additional clinical marker of glucose tolerance.

### Study Strengths and Limitations

The strength of our study is an integrated approach that most studies lack, namely a simultaneous assessment of glucose and insulin kinetics as well as microvascular reactivity and HRV 30 min after OGTT. We acknowledge several limitations. One of the limitations is a rather small sample size, rendering direct evaluation of the potential impact of glucose tolerance questionable. Nevertheless, compared to other studies, our sample was even larger, as the majority included only ten subjects. Gender stratification and/or and sex hormone assessment would be desirable since female sex hormones significantly affect the microvascular response. Yet, by including only females who were in the early follicular phase, we could partially overcome this doubt.

Additional limitation is the use of tests for ANS activity and vascular endothelial function, which were rather indirect and thus did not provide a proper insight into the physiological mechanisms behind them.

It is important to note that HRV spectra as a measure of the ANS activity must be interpreted with caution, as the interpretation of the standard HRV parameters (LF and HF spectral power and the LF/HF ratio) has often been criticised under the claim that it does not directly reflect the ANS activity but rather the modulation of ANS by cardiovascular reflexes, i.e., baroreceptor reflex (LF) and respiratory sinus arrythmia (HF) [48,49,50,51,53]. Using other complementary methods, such as measuring muscle sympathetic neural activity [18,65] or assessing electrodermal activity (EDA), reflecting changes in cutaneous sympathetic activity related to cognitive or emotional stress [66] might increase the reliability of our results [18,65]. Nevertheless, in vivo assessment of ANS activity is difficult, and accordingly, HRV remains the gold standard for indirect assessment of the ANS activity also in the clinics.

Intended to be as non-invasive as possible, the control protocol with water load seemed to be a provocation itself, as it induced subtle alterations in some haemodynamic variables potentially attributable to volume load and elicited physiological reflexes, such as increased sympathetic vasoconstrictor activity and cardiac vagal tone [67]. This stresses the importance of the control trial on one hand but on the other may render some evidence less supportive. Water ingestion has been associated with increased SNS [65] similarly as shown in our study, which is believed to depend on the osmolality of ingested fluid [67]. To avoid the potential problem of hypo-osmolarity, performing a positive control experiment using mannitol solution instead of glucose would be desirable. Indeed, Hoffmann et al. reported increased forearm blood flow after injection of both dextrose and mannitol solutions of the same osmolality, whereas no effect was observed after saline injection [18]. Accordingly, the SNS changes measured in our study could be the result of decreased systemic vascular resistance due to increased osmolality (and not actively induced by glucose per se), although probably of a minimal order of magnitude (as our probands only drank 250 mL of solution). An improvement of our study would therefore be to compare the same effects following iso-osmolar mannitol ingestion, which was not feasible, as mannitol is known to induce osmotic diarrhoea [68]; furthermore, its absorption in the gastrointestinal tract is questionable. It could also be that the observed alterations in the ANS activity were the consequence of psychological stress, as laying supine still for one hour may be a stressor large enough to provoke changes in the ANS balance [66].

In our study, we simulated a postprandial state by performing OGTT. We could obtain a better insight into a corresponding physiological response of SNS and vascular reactivity to acute hyperglycaemia if we extended the study by using protein-rich fluid in addition to OGTT or simply by having subjects eat a well-controlled meal [69]. Including additional biochemical analyses such as, i.e., some markers of inflammation and oxidative stress, might strengthen the results [70]. Indeed, Parker et al. showed that a combined meal induces microvascular impairment in skeletal muscle despite an increase in central haemodynamic measures [64]. Since skin blood flow regulation differs, similar studies in skin microcirculation are warranted.

Taken together, one of the original findings of the present study is that even in healthy subjects with normal glycaemic control, glucose tolerance might be regarded as an important determinant of the SNS and vascular reactivity after OGTT. As the clinical standard for determining glucose tolerance is c_glc_ two hours after OGTT when c_glc_ has already normalised in healthy subjects, establishing a more sensitive measure of glucose tolerance in healthy subjects would be desirable [56,71]. Additional studies assessing similar haemodynamic parameters should be repeated in different time frames after OGTT with a simultaneous assessment of c_glc_ or c_ins_.

## 5. Conclusions

In healthy humans, OGTT slightly impacts SNS activity as assessed by HRV analysis and modestly impairs microvascular reactivity in the volar forearm as measured 30 min after glucose load. Both effects are significantly associated with glucose tolerance. The SNS attenuation presumably reflects feeding-related vagal reflexes, which seem to be more pronounced in subjects with higher glucose tolerance. The impairment of vascular reactivity is only evident in lower glucose tolerance and might include endothelium-mediated and other local mechanisms. Even in the healthy, it is glucose tolerance that determines vascular reactivity shortly after OGTT, exposing the need to develop stronger tools/markers for a timely detection of predisposed individuals. We hope that our study will contribute to a better understanding of the underlying physiological changes following oral glucose load and, possibly, reveal pathological implications in diabetic vascular disease.

## Figures and Tables

**Table 1:** Baseline haemodynamic variables prior to OGTT or water protocol.

	Median (Q1–Q3)
Resting	
RR (ms)	968 (921–995)
SP (mmHg)	115.0 (108.0–122.0)
MP (mmHg)	81.5 (77.5–87.6)
DP (mmHg)	65.7 (60.1–69.4)
PP (mmHg)	51.6 (43.9–54.8)
LD_rest-fp_ (PU)	244 (174–329)
LD_rest-vf_ (PU)	5.9 (4.7–7.5)
LF (n.u.)	34.8 (21.3–54.9)
HF (n.u.)	54.0 (41.5–66.5)
LF/HF	0.57 (0.34–1.33)
PORH, volar forearm	
LD_peak_ (PU)	41 (30–50)
LD_base_ (PU)	6.3 (5.3–10.7)
t_peak_ (s)	10.6 (6.8–12.1)
AUC (PU^2^)	941 (694–1138)
PORH, finger pulp	
LD_peak_ (PU)	336 (281–454)
LD_base_ (PU)	196 (153–258)
t_peak_ (s)	25.8 (18.5–39.6)
AUC (PU^2^)	4477 (2261–11,824)

Data are presented as median and interquartile range (Q1–Q3); N = 20. RR, the duration of the R-R interval of the ECG signal; DP, diastolic digital artery blood pressure; MP, mean blood pressure; SP, systolic blood pressure; PP, pulse pressure; LF, low-frequency spectral power; HF, high-frequency spectral power, LH/HF, ratio of the corresponding spectra; LD_rest-fp_, resting laser Doppler flux on the finger pulp; LD_rest-vf_, resting laser Doppler flux on the volar forearm; PU, perfusion units; n.u., normalised units. See Section 2.6 for further abbreviations of PORH (post-occlusion reactive hyperaemia).

**Table 2:** Haemodynamic variables after OGTT and water protocol in resting conditions and after post-occlusive reactive hyperaemia (PORH).

	OGTT	Water	*p*	CI (95%)
Resting				
ΔRR (ms)	−16 (−50; 6)	26 (−26; 57)	0.120	−58; 8
ΔSP (mmHg)	10.2 (−0.5; 18.7) ^§^	11.6 (6.9; 16.3) ^§^	0.284	−9.6; 3.8
ΔMP (mmHg)	4.1 (−2.9; 9.1)	6.4 (4.3; 11.0)	0.145	−8.6; 1.5
ΔDP (mmHg)	0.7 (−3.6; 5.6)	6.2. (2.3; 7.2) ^§^	0.045 *	−7.9; −0.2
ΔPP (mmHg)	7.3 (3.3; 11.7) ^§^	5.9 (1.4; 9.2) ^§^	0.890	−3.2; 5.0
ΔLD_rest-fp_ (PU)	−54 (−117; −11) ^§^	−56 (−74; −17) ^§^	0.734	−45; 40
ΔLD_rest-vf_ (PU)	0.7 (−0.5; 1.9)	0.5 (−0.3; 1.0)	0.304	−0.6; 3.1
ΔLF (n.u.)	2.4 (−3.1; 13.0)	7.2 (2.2; 14.2) ^§^	0.022 *	−17.1; −1.0
ΔHF (n.u.)	−2.3 (−11.3; 3.5)	−8.3 (−13.3; −1.9) ^§^	0.107	−1.3; 10.3
ΔLF/HF	0.05 (−0.17; 0.40)	0.205 (0.03; 0.62) ^§^	0.022 *	−0.93; −0.04
PORH, volar forearm				
ΔLD_peak_ (PU)	−1 (−4; 6)	3 (−1; 6)	0.485	−8; 3
ΔLD_base_ (PU)	0.2 (−0.7; 1.2)	0.3 (−0.7; 1.2)	1.000	−1.1; 1.5
Δt_peak_ (s)	−0.2 (−2.2; 3.6)	−1.2 (−2.3; 0.4) ^§^	0.119	−0.6; 4.9
ΔAUC (PU^2^)	97 (−146; 243)	126 (−104; 246)	0.442	−171; 369
PORH, finger pulp				
ΔLD_peak_ (PU)	−32 (−70; −9) ^§^	−18 (−37; 4)	0.071	−74; 3
ΔLD_base_ (PU)	−13.0 (−53.1; 3.4) ^§^	−34.2 (−73.1; −22.1) ^§^	0.468	−85.0; 39.8
Δt_peak_ (s)	1.2 (−5.7; 20.8)	5.4 (−7.2; 14.0)	0.734	−15.4; 23.2
ΔAUC (PU^2^)	2170 (−813; 3932)	962 (−1786; 3528)	0.963	−3812; 3955

Data are presented as median and interquartile range; N = 20. Δvariable, post-treatment–pre-treatment (holds for all variables listed); LF, low-frequency spectral power; HF, high-frequency spectral power; LH/HF, ratio of the corresponding spectra; LD_rest-fp_, resting laser Doppler flux measured on the finger pulp; LD_rest-vf_, resting laser Doppler flux measured on the volar forearm; LD_peak_, LD_base_, and t_peak_, AUC–PORH parameters (refer to Methods section); PU, perfusion units; n.u., normalised units; CI, confidence interval. ^§^ post-provocation vs. pre-provocation, Wilcoxon test; * *p* ˂ 0.05 Wilcoxon test, OGTT vs. water change.

**Table 3:** Linear regression of post-occlusive reactive hyperaemia (PORH) and heart rate variability (HRV) parameters with respect to glucose concentration increase after OGTT.

	r	*p*
HRV		
LF	0.488	0.040 *
HF	−0.528	0.024 *
LF/HF	0.542	0.020 *
PORH, volar forearm		
ΔLD_peak_	−0.619	0.004 *
Δt_peak_	−0.196	0.408
ΔAUC	−0.408	0.074
PORH, finger pulp		
ΔLD_peak_	−0.126	0.597
Δt_peak_	0.012	0.961
ΔAUC	−0.358	0.132

r, Pearson’s correlation coefficient; N = 20; * *p* ˂ 0.05, Pearson’s test. See Section 2.6 for abbreviations description.

**Table 4:** Plasma glucose and serum insulin concentration change with respect to fasting values following oral glucose load.

Time (min)	Δc_glc-VB_ (mM)	*p*	Δ_cins-VB_ (µUml^−1^)	*p*
30	1.7 ± 1.3	0.017 *	20.2 ± 21.7	0.007 *
50	1.5 ± 1.7	0.034 *	28.1 ± 28.9	0.000 *
90	0.9 ± 1.2	0.560	32.6 ± 21.5	0.000 *

Data are presented as means ± SD (standard deviation). Δc_glc-VB_, change in plasma glucose concentration, assessed in venous blood samples (VB) with respect to fasting value; Δc_ins-VB_, change in serum insulin concentration with respect to fasting value; * *p* ˂ 0.05, repeated-measures ANOVA, post hoc Tukey test.

## Data Availability

The data presented in this study are available on request from the corresponding author.
